# Quantifying Variation in Gait Features from Wearable Inertial Sensors Using Mixed Effects Models

**DOI:** 10.3390/s17030466

**Published:** 2017-02-25

**Authors:** Kellen Garrison Cresswell, Yongyun Shin, Shanshan Chen

**Affiliations:** Department of Biostatistics, Virginia Commonwealth University, Richmond, VA 23298, USA; cresswellkg@mymail.vcu.edu (K.G.C.); yongyun.shin@vcuhealth.org (Y.S.)

**Keywords:** pervasive gait analysis, mounting location uncertainty, gait speed variation, gait data quality, sources of variation, gyroscope, accelerometer, statistical characterization, random effects models, fixed effects models

## Abstract

The emerging technology of wearable inertial sensors has shown its advantages in collecting continuous longitudinal gait data outside laboratories. This freedom also presents challenges in collecting high-fidelity gait data. In the free-living environment, without constant supervision from researchers, sensor-based gait features are susceptible to variation from confounding factors such as gait speed and mounting uncertainty, which are challenging to control or estimate. This paper is one of the first attempts in the field to tackle such challenges using statistical modeling. By accepting the uncertainties and variation associated with wearable sensor-based gait data, we shift our efforts from detecting and correcting those variations to modeling them statistically. From gait data collected on one healthy, non-elderly subject during 48 full-factorial trials, we identified four major sources of variation, and quantified their impact on one gait outcome—range per cycle—using a random effects model and a fixed effects model. The methodology developed in this paper lays the groundwork for a statistical framework to account for sources of variation in wearable gait data, thus facilitating informative statistical inference for free-living gait analysis.

## 1. Introduction

In recent years, wearable inertial sensors have emerged as a promising tool for gait analysis, with the potential to provide continuous assessment of gait-associated disorders and diseases in free-living environments [1]. Medical applications range from the assessment of neurological or neuropathic disorders [2,3,4,5,6] and fall risks [7,8,9,10], to evaluating the efficacy of prosthetics and orthoses [11,12,13]. Assessment of these disorders and diseases can greatly benefit from the continuous out-of-lab assessment of gait. Ideally, effective use involves sending the sensors to patients’ homes, and patients will simply strap the sensors around their arm or leg (following instructions from the medical researchers or caregivers), and wear the sensors as they go about their everyday life.

However, the freedom of having patients essentially self-administer the test comes with certain challenges. One major challenge is that such flexibility introduces sources of variation that can greatly impact gait data quality, such as mounting uncertainty and physiological variation in the free-living environment [1]. For example, while gait speed is often considered an important feature in predicting frailty in the elderly [7] and assessing gait pathologies [14], it is also intertwined with physiological variation due to mood, types of shoe, terrain, and energy levels, among other factors [15,16,17]. Meanwhile, with the current strap-on method of mounting sensors, the sensors can at times move around body segments without detection. Since inertial sensors are sensitive to orientation changes, mounting location shifts can also cause significant variations in sensor data. While these issues can be mitigated in a controlled laboratory environment by adopting elaborate calibration procedures [18] before and after data collection, it is very challenging to control or correct these factors in the free-living environment during continuous data collection. Therefore, these factors must be characterized by statistical modeling before the gait analysis results can be applied to assist clinical inference.

The first step to achieving this is to properly identify sources of variation and quantify their impact on gait data. While we currently know that factors such as speed and mounting location can contribute to changes in sensor readings, the degree to which each factor drives variation has not been statistically established. Therefore, in this paper, we propose a statistical framework for decomposing and quantifying sources of variation. First, we collect gait data by conducting a full-factorial experiment with 48 factorial combinations of four commonly identified sources of variation. Secondly, we analyze the gait data using a random effects model and a fixed effects model to quantify sources of variation in one gait outcome (range per gait cycle), elaborating the methodology and demonstrating the viability of the proposed method. Lastly, we discuss the results from this statistical framework and their implications for wearable sensor-based gait analysis.

## 2. Related Work

Previous work has sought to identify and remedy errors and variation due to sensor mounting uncertainty from an engineering perspective. Chen et al. devised a simple procedure—applied prior to data collection—that aligns the inertial frame to the global frame and remedies mounting errors [19]. To mitigate mounting shifts, they used an elastic strap with a tennis grip to maximize the friction. Amini et al. developed an autonomous method to determine the mounting location on the body for activity recognition [20]. However, their method can only identify different body segments, rather than the finer location within a body segment, which is required for high-precision gait analysis [20]. Seel et al. devised a method that allows for arbitrary mounting locations for joint knee angle calculation [21]. Although this method is effective and practical, it does not address the issue of mounting location shift and is only applicable to that particular gait outcome. In general, these methods are limited to providing accurate one-time calibration. Even with proper pre- and post-calibration to detect mounting location shifts [18], these shifts can still be unnoticed during continuous data collection in day-to-day use, despite proper precautions to prevent sensor shift.

Besides mounting location, gait speed is another uncontrollable source of variation that can affect gait analysis. In the laboratory setting, gait speed is fairly easy to measure using a treadmill or a stop watch and tape measures, and can then be adjusted for in post analysis. However, these conditions do not exist in the natural setting. Since it is difficult for inertial sensors to measure accurate spatial information for an extended period [19], it is inappropriate to rely on inertial sensors to provide ground truth gait speed information in a free-living situation.

Therefore, for real-world deployment, researchers must be ready to accept the uncertainties that come with wearable sensor-based gait analysis. Although the uncertainties cannot be easily controlled, they can be identified, characterized, and modeled using statistical methods. The quantified variation can help researchers determine the sample size required through statistical power analysis and identify variables that need to be accounted for during data analysis. By moving the burden of dealing with variation onto analysis, we avoid the use of ad-hoc correction algorithms that may distort raw data, and encourage a more communicable biostatistical framework to account for intra-/inter-subject gait variability.

## 3. Experimental Design

Our experiment employed four Shimmer3 sensor nodes (Shimmer Sensing, Doublin, Ireland). Each sensor node consists of a three-axis accelerometer, a three-axis gyroscope, and a three-axis magnetometer. Both accelerometer and gyroscope sensors were manually calibrated with the aid of Shimmer 9 Degrees of Freedom (9 DOF) Calibration software prior to data collection [22].

### 3.1. Full-Factorial Setup

**Figure 1:**
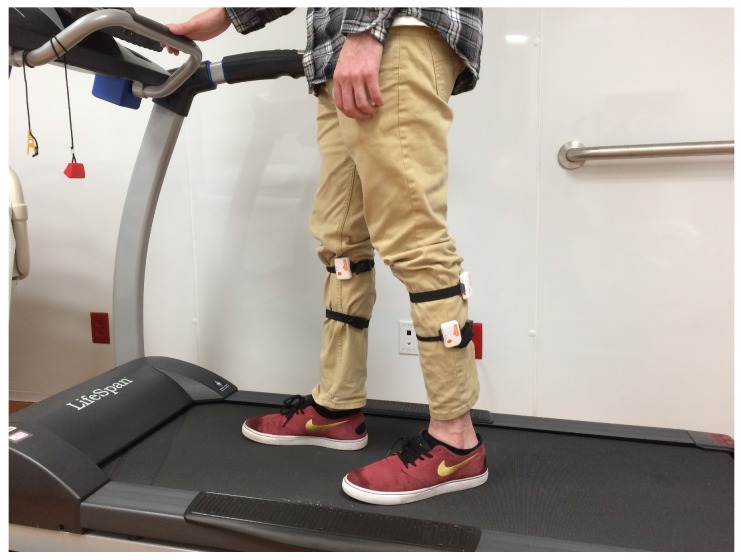
Experimental setup: a single subject getting ready for full-factorial trials on a treadmill.

To identify factors that may potentially contribute to sensor variability, a single-subject full-factorial experiment was designed and conducted, as shown in Figure 1. Four factors of interest were examined: mounting location, mounting leg, sensor, and speed. These factors were chosen due to their relative importance in obtaining an accurate sensor reading. The factorial design ensures that each combination of the factors is tested, which enables us to test how each factor contributes to total variation and compare interaction effects between factors.

**Figure 2:**
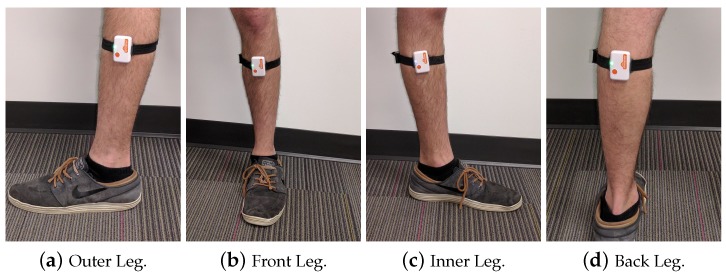
Overview of mounting locations (left leg).

In freestyle walking situation, mounting location can shift around the leg transversely or along the leg longitudinally. In this paper, we focus on investigating the transverse shifts, randomizing the mounting locations to four equidistant extremes: outer, inner, front, and back of the leg, as shown in Figure 2. Additionally, the mounting legs were randomized so that the sensor was mounted on either the left or right leg. Two different sensors were also interchanged to test for differences between them. Gait speed was the final variable randomized and controlled by treadmill, varying at 1.8 mph, 2.4 mph, and 3.0 mph. These speeds were intended to encompass typical walking speeds for healthy human subjects. A third reference sensor was mounted on the outer right leg to provide ground truth data for each session.

In total, 48 factorial combinations were tested from the four sources of variation. For each combination, an approximately one-minute treadmill session was performed. The data was recorded at 120 Hz, totalling 245,277 data points spread across the 48 sessions. Once the collection phase was over, sensor data was cleaned to remove non-walking data and concatenated with the design matrix which contains the indicator variables for each session. Data management was performed in MATLAB^®^.

### 3.2. Random Effects Modeling

Gait variation from the full-factorial design was quantified using a random effects model. The random effects model—also known as a variance component model—can provide a breakdown of the variation contributed by each factor; i.e., the percentage of total variability explained by each factor [23]. Factors with a small percentage are of comparatively less concern in analysis because they contribute less to differences in gait outcomes. On the other hand, a large percentage indicates that special care must be taken to control for this variable in the analysis, and thus during measurement. The model used in this paper is demonstrated by

(1) $$Y_{ijklt}=\mu +\alpha_{i}+\beta_{j}+\gamma_{k}+\delta_{l}+{\left(\alpha \beta \right)}_{ij}+{\left(\alpha \gamma \right)}_{ik}+{\left(\alpha \delta \right)}_{il}+{\left(\beta \gamma \right)}_{jk}+{\left(\beta \delta \right)}_{jl}+{\left(\gamma \delta \right)}_{kl}+\epsilon_{ijklt}.$$

In Equation (1), each individual coefficient corresponds to the amount of variance explained by a given factor. Specifically:

$Y_{ijklt}$ is the gait outcome given some combination of factor levels,
*μ* is the grand mean outcome,
$\alpha_{i}$ is the main effect of the *i*th mounting location,
$\beta_{j}$ is the main effect of the *j*th speed,
$\gamma_{k}$ is the main effect of the *k*th sensor,
$\delta_{l}$ is the main effect of the mounting leg,
$\alpha \beta_{ij}$, $\alpha \gamma_{ik}$, $\alpha \delta_{il}$, $\beta \gamma_{jk}$, $\beta \delta_{jl}$, and $\gamma \delta_{kl}$ are two-way interaction effects
$\epsilon_{ijklt}$ is a random error term capturing unexplainable time-to-time variability, for time index t.

The outcome $Y_{ijklt}$ represents either the y-axis angular velocity ($G_{Y}$) or the x-axis acceleration ($A_{X}$), depending on which outcome is being analyzed. All terms except mu in Equation (1) are normally distributed with respective variances of $\sigma_{\alpha }^{2}$, $\sigma_{\beta }^{2}$, $\sigma_{\gamma }^{2}$, $\sigma_{\delta }^{2}$, $\sigma_{\alpha \beta }^{2}$, $\sigma_{\alpha \gamma }^{2}$, $\sigma_{\alpha \delta }^{2}$, $\sigma_{\beta \gamma }^{2}$, $\sigma_{\beta \delta }^{2}$, $\sigma_{\gamma \delta }^{2}$, and $\sigma_{\epsilon }^{2}$. The physical meanings of the indices are listed in Table 1.

The random-effects model represented by Equation (1) analyzes the main and two-way interaction effects of the four factors in the full-factorial design. These interactions allow us to examine whether one factor affects the gait variability differently across the factor levels of another factor.

### 3.3. Outcome Variables

**Figure 3:**
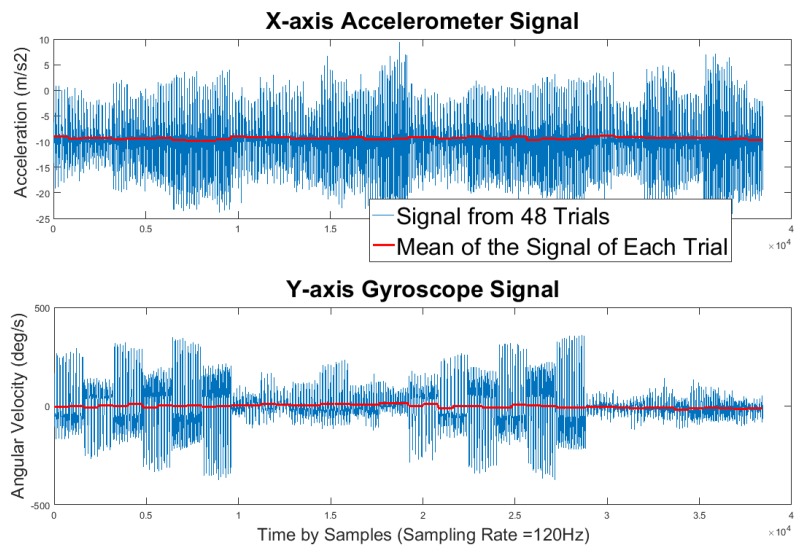
Raw x-axis accelerometer signals and y-axis gyroscope signals from the 48 trials.

The first step to fitting the random effects model is selecting an informative transformation of each outcome variable. While choosing the raw data as the outcome may seem intuitive, inertial sensor signals are mean centered around some constant. The random effects model relies on detecting differences between group means. The consequence is that the mean differences are virtually zero, attributing virtually none of the total outcome variability to the between-group differences, but all to the within-group differences. This manifests itself in a large *ϵ* term, indicating that the majority of the variation is unexplained. The zero-mean phenomenon is captured in Figure 3, where 800 consecutive samples from each trial are displayed along with their means.

**Figure 4:**
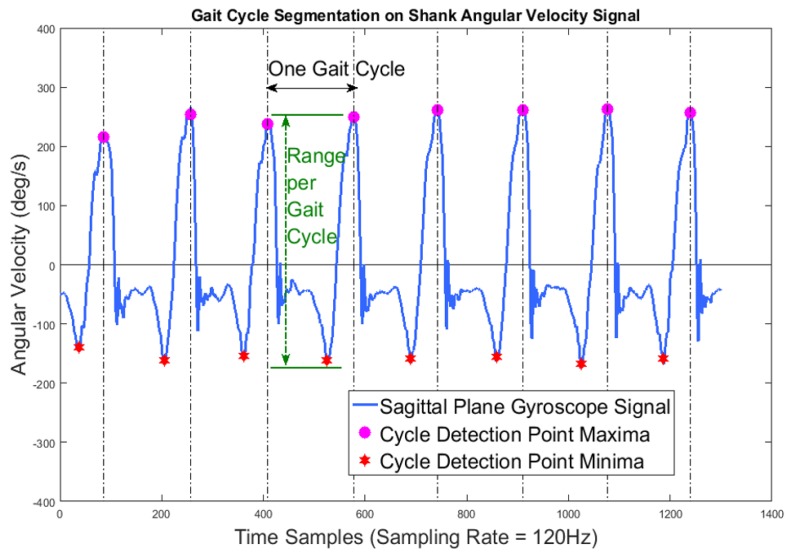
Segmentation of y-axis gyroscope data.

Therefore, we picked a gait feature that transforms the raw data to meaningful positive quantities as our outcome variable $Y_{ijktl}$. To avoid errors introduced by more complex feature extraction algorithms, we picked a statistical gait feature that is resilient to feature extraction error – range per gait cycle. Since gait cycle detection algorithms [19] can accurately segment gait cycles when the temporal resolution of the signal is sufficiently high (e.g., sampling rate of 120 Hz), this feature can be extracted by calculating the difference between the maximum and minimum value in each gait cycle. The cycle segmentation and range calculation process are demonstrated by Figure 4, where purple circles indicate cycle maxima and red stars indicate minima. This range feature can easily be applied to the raw data from all nine axes of a typical 9DOF inertial sensor.

In this paper, we focus on the analysis of two variables: y-axis gyroscope and x-axis accelerometer data, capturing sagittal-plane angular velocity and walking-direction acceleration, respectively, when the sensor is mounted on the outer side of the leg. These two variables are selected due to their straightforward clinical interpretations.

## 4. Results

The results of fitting the random-effects models in Equation (1) are captured in Table 2.

Table 2 lists the total outcome variability explained by the four factors and their interaction terms. Each percentage in the table is the percentage of the variance explained by a given factor relative to the overall variance. The range feature of y-axis gyroscope data ($G_{Y}$) is greatly affected by transverse shifts of mounting location, explaining 83.59% of the total variation. The varying gait speed contributes 4.47% to the the total variation, and the location by speed and location by leg interaction effects explain 5.57% and 3.18%, respectively. The contribution of other factors to the total variation is comparatively very small. The amount of unexplained variance is 2.07%, suggesting that the main and interaction variables explain the vast majority of the variation. For the range feature of x-axis accelerometer data ($A_{X}$), mounting location and speed explain 27.22% and 53.10% of the total variation, respectively. The variability explained by the interaction terms is comparatively trivial. In the case of the accelerometer, 11.70% of the total variance remains unexplained.

To gain a better understanding of the interplay between factors and outcomes, we fit fixed effects models using only two factors: mounting location and speed. These two factors were selected based on Bayesian information criterion (BIC) [24]. Our preliminary analysis shows that speed is linearly related to acceleration and angular velocity, so it is included as a quantitative variable. Mounting location was coded by three different dummy variables, indicating back ($L_{B}$), inside ($L_{I}$), and front locations ($L_{F}$) on the leg. The model is

(2) $$Y_{t}=\beta_{0}+\beta_{1}L_{Bt}+\beta_{2}L_{It}+\beta_{3}L_{Ft}+\beta_{4}\left(S_{t}-1.8\right)+\epsilon_{t},$$

where outcome $Y_{t}$ is either y-axis angular velocity ($G_{Y}$) or x-axis acceleration ($A_{X}$), $L_{B}$, $L_{I}$, $L_{F}$ are dummy variables such that:

$$L_{B}=\left\{\begin{array}{cc} 1, & \mathrm{if}\;\;sensor\;is\;mounted\;on\;the\;back\;of\;the\;leg\\ 0, & \mathrm{otherwise} \end{array}\right.$$

$$L_{I}=\left\{\begin{array}{cc} 1, & \mathrm{if}\;\;sensor\;is\;mounted\;on\;the\;inside\;of\;the\;leg\\ 0, & \mathrm{otherwise} \end{array}\right.$$

$$L_{F}=\left\{\begin{array}{cc} 1, & \mathrm{if}\;\;sensor\;is\;mounted\;on\;the\;front\;of\;the\;leg\\ 0, & \mathrm{otherwise}. \end{array}\right.$$

If the sensor is mounted on the outer portion of the leg, all dummy variables will be 0. $S_{t}$ is the quantitative speed (i.e. 1.8 mph, 2.4 mph and 3 mph), centered at the lowest speed. Therefore, the intercept $\beta_{0}$ represents the mean outcome at the lowest speed, 1.8 mph, with outer leg mounting.

The results are summarized in Table 3 for both the accelerometer and the gyroscope, with fixed effect estimates followed by their standard errors in parentheses.

Examining the range feature from the y-axis gyroscope data, we see that relative to outer leg mounting, back and front leg mounting locations had large negative gaps in their mean ranges of angular velocity (−310.96 and −314.48, respectively); in contrast, inner leg mounting had a modest positive gap in mean range (8.06), controlling for the speed. Speed was positively associated with the gyroscope outcome (67.35), controlling for the mounting location.

The impact of inner leg mounting on the range of acceleration was not significantly different from outer leg mounting (−0.04), controlling for the gait speed. On the other hand, with controlled gait speed, the back and front leg mountings had positive (5.27) and negative (−1.50) gaps in comparison to outer leg mounting, respectively. With perfect mounting, it is expected that front and back leg mounting should produce virtually identical coefficients, as is the case between inner and outer leg mounting. Our findings indicate that this is not the case. The data suggest mounting location induces extra variability in the range of acceleration. Naturally, speed is positively associated with the range of motion, controlling for the mounting location.

## 5. Discussion

Our proposed models and experimental results shed light on the breakdown of uncertainty in wearable sensor-based gait data, using range per cycle as an example of gait outcomes. The results of the random-effects model reveal that of the four main factors and their two-way interactions, mounting location shift accounts for 27.22% and 83.59% of the total variability in walking-direction acceleration and sagittal plane angular velocity respectively, while gait speed accounts for 53.10% and 4.47% of the total variability. Analysis of the fixed effects model (2) reveals that the gait variability from the mounting location can be substantially reduced by avoiding front and back leg mounting locations.

For walking-direction accelerometer data, the variation of the gait range feature is mostly influenced by gait speed. In the natural setting, this varies due to uncontrollable factors such as mood and energy levels, and is challenging to measure accurately. Therefore, when analyzing gait in freestyle walking, range-related features extracted from walking-direction accelerometer data should be analyzed with care, adjusting for gait speed. For y-axis gyroscope data, the variation is mostly influenced by mounting location—a factor that is controllable through good mounting practice. Although ideally, mounting location should not vary, it is difficult to achieve in reality. So if the mounting location has to vary, it is better for it to vary between inner and outer leg mounting locations. Since we used extreme mounting shift to characterize this factor, we expect the impact of this factor on both gyroscope and accelerometer data to be smaller in reality. It is interesting to note that for both outcome variables, little variation is contributed by changing sensors or by swapping sensors between legs. This means that with proper sensor calibration prior to data collection, the gait data variation from using different sensor nodes is negligible. Furthermore, for healthy subjects with symmetric gait, a single inertial sensor node is sufficient to characterize their bilateral gait patterns.

The results of the fixed effects models highlighted the discrepancies between front–back mounting versus inner–outer mounting, shown by the differences in the coefficients between the four mounting locations in Table 3. While mounting on the inner and outer shank show similar effects on the range feature, the vast difference in range between these combinations suggests that mixing front or back mounting with either inner or outer mounting would result in poor-quality data. This is due to the drastic mismatch between the latent measurement and the assumed sensing axes when mounting location is shifted from the side of the shank to the front or back of the shank. In other words, when mounted on the side of the shank, the gyroscope axis capturing the sagittal plane motion will instead capture frontal plane motion once shifted to the front or back of the shank. A similar issue occurs with accelerometers shifting away from walking-direction acceleration and measuring the acceleration of lateral leg movement instead.

Our random effects model explains about 98% of the variation in gyroscope data and 88% of the variation in accelerometer data. The unexplained variation can be caused by a number of factors. For example, during data collection in our full-factorial experiment, we did not consider the time of day or the mood of the subject, which can affect gait patterns—especially in free-living gait analysis and/or in a pathological population [25]. Another less observable but more persistent factor may be the subtle shifting of mounting location during walking, especially when mounted on a less flat surface such as the bony front or muscular back of the shank. It is also interesting to note that the unexplained variation in the accelerometer data is higher than in the gyroscope data. Since there may be a subtle alignment difference between the same assigned mounting locations during 48 trials, and accelerometer data are more sensitive to subtle changes in orientation than gyroscope data, this could cause comparatively more unexplained variation in accelerometer data.

This pilot study focused on methodology development, and thus the current experiment only concerns a single, healthy, non-elderly subject. Moreover, we did not conduct an exhaustive investigation into multiple gait outcomes and all possible sources of variation. Further research on subjects with pathological gait patterns may lead to different percentages of variation. For instance, data from a patient with an asymmetric gait will likely exhibit more variation caused by moving the sensor between legs than for someone with a symmetric gait. This paper also focuses on four extreme mounting locations in order to assess the range of mounting effects so that further studies involving multiple subjects can leverage this information for statistical power calculation. In reality, this variation may be less severe.

In the future, we will extend this method to intra-/inter-subject variability in more gait features, with consideration of individual characteristics such as gender, ethnicity, age, height, and weight. The framework developed can also be extended to other types of sensors for gait measurement, such as insole sensors [26] and ear-worn sensors [27]. The results of our future work will reveal a complete picture of variations in human gait data collected by wearable sensors, and eventually prepare wearable-sensor based gait analysis for real-world clinical assessment and diagnosis.

## 6. Conclusions

This paper proposed a framework for quantifying variation in gait data collected by wearable inertial sensors using mixed effects models. Utilizing a randomized controlled trial on a healthy, non-elderly subject with a full-factorial design of 48 factor combinations, we characterized how four common factors and their interactions influence a gait feature derived from wearable inertial sensors. Our analysis demonstrated that mounting location and gait speed are the two dominant factors contributing to a large percentage of the variation in both accelerometer (80.32%) and gyroscope (88.06%) data, while the variability from using different sensors (after calibration) and swapping the mounting leg (provided the gait being assessed is symmetric) is negligible. Future work will extend the method to other sensor axes, gait features and multiple subjects to characterize intra/inter-subject variability.

## Figures and Tables

**Table 1:** Identified sources of variation in factorial design.

Factor *α*		Factor *β*		Factor *γ*		Factor *δ*	
i	Mounting Location	j	Speed	k	Sensor Node	l	Mounting Leg
0	Outer Leg	0	1.8 mph	0	Sensor Node 1	0	Left Leg
1	Back Leg	1	2.4 mph	1	Sensor Node 2	1	Right Leg
2	Inner Leg	2	3.0 mph				
3	Front Leg						

**Table 2:** Percentages of variance explained in range data.

Source	$G_{Y}$	$A_{X}$
*α* (Location)	83.59%	27.22%
*β* (Speed)	4.47%	53.10%
*γ* (Sensor)	0%	0%
*δ* (Leg)	0%	0%
αβ	5.57%	2.56%
αγ	0.66%	0.03%
αδ	3.18%	1.23%
βγ	0.03%	1.33%
βδ	0.43%	2.50%
γδ	0%	0.34%
Unexplained (*ϵ*)	2.07%	11.70%

**Table 3:** Analysis of linear regression models.

Source	Coefficient	$G_{Y}$	$A_{X}$
Outer Leg (Intercept)	$\beta_{0}$	448.87 (3.24)	16.49 (0.13)
Back Leg ($L_{B}$)	$\beta_{1}$	$-310.96{\;}^{**}$ (3.89)	$5.27{\;}^{**}$ (0.15)
Inner leg ($L_{I}$)	$\beta_{2}$	$8.06{\;}^{*}$ (3.80)	−0.04 (0.15)
Front Leg ($L_{F}$)	$\beta_{3}$	$-314.48{\;}^{**}$ (3.70)	$-1.50{\;}^{**}$ (0.15)
Speed (S)	$\beta_{4}$	$67.35{\;}^{**}$ (2.86)	$6.80{\;}^{**}$ (0.11)

Note: * indicates *p*-value < 0 .05 and ** indicates *p*-value < 0.01.
